# Dietary Isorhamnetin Alleviates Alcoholic Liver Injury in Mice by Regulating Gut Microbiota via the Gut–Liver Axis

**DOI:** 10.3390/nu18183062

**Published:** 2026-09-19

**Authors:** Lulu Wu, Yun Bai, Erzheng Su, Fuliang Cao, Xianying Fang, Linguo Zhao

**Affiliations:** 1 State Key Laboratory for Development and Utilization of Forest Food Resources, Nanjing Forestry University, Nanjing 210037, Chinaxianyingfang@njfu.edu.cn (X.F.); 2 Jiangsu Co-Innovation Center of Efficient Processing and Utilization of Forest Resources, College of Chemical Engineering, Nanjing Forestry University, Nanjing 210037, China; 3 College of Light Industry and Food Engineering, Nanjing Forestry University, Nanjing 210037, China; 4 Co-Innovation Center for Sustainable Forestry in Southern China, Nanjing Forestry University, Nanjing 210037, China; 5 Jinpu Research Institute, Nanjing Forestry University, Nanjing 210037, China

**Keywords:** isorhamnetin, alcoholic liver disease, gut–liver axis, gut microbiota, anti-inflammation, natural active products

## Abstract

**Background:** Alcoholic liver disease (ALD) refers to a progressive liver injury syndrome induced by chronic excessive alcohol intake. Current treatment options are mainly limited to symptom management, and there are safety risks associated with long-term use. Hence, there is an urgent demand to discover new therapeutic targets and investigate natural active substances. Isorhamnetin (IS) is an O-methylated flavonoid, and its specific structure confers unique biological activities and relatively good oral bioavailability, making it a potential natural candidate for the prevention of metabolic liver diseases. However, few studies have explored the application and action mechanism of IS in the intervention of alcoholic liver injury and intestinal flora imbalance. **Methods:** This work established a mouse model of chronic alcoholic liver injury to evaluate the beneficial effects of IS on hepatic injury, oxidative stress, and intestinal flora disturbance. **Results:** The results showed that IS intervention therapy can alleviate the symptoms of increased liver index, ALT, AST, TC, and TG levels due to alcohol intake. Furthermore, IS can alleviate liver oxidative stress caused by alcohol consumption, thereby protecting the liver of mice. Intestinal analysis in mice revealed that IS elevated the expression of tight junction proteins ZO-1 and Claudin-1, thereby attenuating alcohol-induced intestinal hyperpermeability and LPS translocation. This further suppressed the activation of the hepatic LPS/TLR4 signaling pathway and decreased the production of TNF-α and IL-1β. Meanwhile, IS significantly improved the intestinal flora disorder induced by alcohol in a dose-dependent manner. Compared with the EtOH group, IS treatment, particularly at the high dose (EISH), significantly increased the Ace, Chao1, Shannon, and Simpson indices. **Conclusions:** Consistently, NMDS analysis based on the OTU level showed that the IS-treated groups clustered away from the EtOH group and shifted toward the Control group, indicating a partial restoration of the overall microbial community structure toward that of healthy controls. In terms of species composition, IS treatment enriched beneficial microbial communities and inhibited the proliferation of pathogenic bacteria, efficiently restoring intestinal flora homeostasis. Collectively, these findings suggested that the hepatoprotective effects of IS were closely associated with the amelioration of gut microbiota dysbiosis and intestinal barrier function, pointing to a potential involvement of the gut–liver axis, although its causal contribution remains to be confirmed by further mechanistic studies (e.g., microbiota depletion or fecal microbiota transplantation). The findings not only provide new scientific evidence for the hepatoprotective effects of isorhamnetin but also offer a preliminary theoretical basis for the development of natural products potentially targeting the gut–liver axis.

## 1. Introduction

Alcoholic liver disease (ALD) is a progressive hepatic injury syndrome caused by long-term heavy alcohol intake, and it stands as one of the leading causes of liver disease-related mortality worldwide [1,2]. According to statistics from the World Health Organization (WHO), approximately 3.8% of deaths worldwide are directly attributable to alcohol use, with ALD accounting for more than 50% of alcohol-related liver injury cases. The pathological progression of ALD encompasses fatty degeneration, hepatitis, fibrosis, and ultimately cirrhosis and liver cancer [3,4]. Chronic excessive alcohol consumption disrupts the balance between hepatocyte injury and repair by inducing oxidative stress, inflammatory responses, and metabolic disturbances [5]. The current clinical treatment strategies for alcoholic liver injury primarily focus on pharmacological intervention and dietary modulation, aiming to alleviate the pathological process and restore liver-gut homeostasis [6,7]. However, existing medications are largely limited to symptomatic treatment, and long-term use poses safety risks, necessitating the urgent exploration of novel intervention targets and natural active ingredients. In recent years, the function of the gut–liver axis in the occurrence and development of liver diseases has attracted extensive interest: disrupted intestinal microbiota, damaged intestinal barrier integrity, and abnormal translocation of gut-derived metabolites such as lipopolysaccharide and short-chain fatty acids can directly exacerbate hepatic inflammation and metabolic imbalance through the portal vein circulation. Consequently, targeting the regulation of gut–liver axis homeostasis offers a novel approach for the prevention and treatment of ALD [8,9].

Flavonoids possess excellent antioxidant, anti-inflammatory, and intestinal flora regulatory capacities, which enables them to serve as effective natural protective reagents against metabolic liver-related diseases [10,11]. Thus, it is of great significance to develop flavonoid active monomers with liver-protection and sobering-up effects in plants and investigate their mechanisms of action. Studies have shown that isorhamnetin is widely found in the fruits and leaves of plants, such as *Hippophae rhamnoides*, *Ginkgo biloba*, *Crataegus pinnatifida*, and *Lycium barbarum* (Figure S1), which can alleviate alcohol-induced oxidative damage through multiple pathways [12,13]. Notably, isorhamnetin (IS), an important methylated derivative of quercetin, features a methoxy group at the C3′ position in its structure, significantly enhancing its lipophilicity and biomembrane permeability, which may improve its oral bioavailability [14]. Research has demonstrated that isorhamnetin can mitigate oxidative damage by activating the Nrf2 pathway and alleviate inflammatory responses by inhibiting the NF-κB signaling pathway [15]. However, its protective role in alcoholic liver injury, especially the mechanisms regulated through the gut–liver axis, has not been systematically elucidated. Furthermore, the specific targets of its action, the gut microbiota-host metabolism interaction network, and its clinical translation potential require further exploration.

Based on the well-established evidence that chronic alcohol intake compromises intestinal barrier integrity, induces gut dysbiosis, and promotes LPS translocation to activate the hepatic TLR4 signaling pathway [8,9], together with the documented capacity of flavonoids to modulate the gut microbiota and repair the intestinal barrier [10,11], we hypothesized that isorhamnetin may exert its hepatoprotective effect through the gut–liver axis. As this specific mechanism has not been previously investigated for isorhamnetin, the present study is thus both evidence-informed and exploratory in nature and is designed to elucidate how isorhamnetin disrupts the vicious cycle of “intestinal leakage-hepatic inflammation” via repairing the integrity of the intestinal barrier, modulating gut microbial composition, and inhibiting the activation of the TLR4/MyD88 signaling pathway in the liver. The findings of this study not only provide new scientific evidence for the hepatoprotective effects of isorhamnetin but also offer theoretical support for the development of natural products targeting the gut–liver axis.

## 2. Materials and Methods

### 2.1. Materials and Antibodies

Isorhamnetin was prepared in our laboratory through enzymatic conversion (purity > 98%) (the specific preparation method and the mass spectra of the product are shown in Figures S2 and S3) [16]. TLR4 primary antibodies were obtained from Proteintech (Chicago, IL, USA), and myeloid differentiation factor 88 (MyD88) primary antibodies were sourced from Abcam (Cambridge, MA, USA). The primary antibodies against phosphorylated NF-κBp65 (p-NF-κBp65) were obtained from Cell Signaling Technology (Boston, MA, USA).

### 2.2. Animals and Experimental Design

The study utilized six-week-old male C57BL/6J mice sourced from Beijing Vital River Laboratory Animal Technology Co. Ltd., located in Beijing, China. In female animals, sex hormone levels (particularly estrogen) fluctuate with the estrous cycle, which can significantly affect alcohol-metabolizing enzyme activities, hepatic lipid metabolism, and inflammatory responses, thereby introducing uncontrollable variability. To exclude this confounding factor and ensure the stability of the modeling, a single sex was employed in this study. These mice were raised under controlled conditions with consistent temperature (22 ± 2 °C) and humidity (50 ± 10%), a regular 12 h light-dark cycle, and ad libitum access to food and water. The ethical treatment of all animals was in accordance with the standards outlined in the Guide for the Care and Use of Laboratory Animals, published by the U.S. National Institutes of Health. To minimize pain and distress, animals were housed under standard conditions with environmental enrichment and had ad libitum access to food and water. No unexpected adverse events were observed during the study. Pre-defined humane endpoints were established. Animal welfare was monitored at least twice daily by trained personnel using a clinical score sheet. Any animal reaching these endpoints would be immediately humanely euthanized. No animal reached a humane endpoint during the course of the study. All experiments were conducted in the SPF-grade Animal Experiment Center of Nanjing University under a rental arrangement. The animal study protocol was reviewed and approved by the Institutional Animal Care and Use Committee of Nanjing Forestry University (Approval No. 2024016). All animal experiments were conducted in accordance with the UK Animals (Scientific Procedures) Act 1986, as amended in 2012 to implement Directive 2010/63/EU, and complied with the ARRIVE guidelines 2.0.

After one week of acclimation, the mice (*n* = 8 per group) were randomly assigned to four groups using a random number table generated by SPSS software (IBM Corp., Armonk, NY, USA), ensuring comparable mean body weights across groups. During sample collection and all subsequent histological and immunohistochemical evaluations, investigators were blinded to group allocation: samples were coded by a researcher not involved in the assessment, and the codes were not revealed until the completion of data analysis. The mice in these four groups were then subjected to different feeding conditions for ten days. The Control group was fed the Lieber DeCarli control liquid diet. The ethanol (EtOH) group was fed the Lieber DeCarli ethanol liquid diet. The ethanol + low-concentration IS (EISL) group was fed the Lieber DeCarli ethanol liquid diet and received IS (50 mg/kg) via oral gavage. The ethanol + high-concentration IS (EISH) group was fed the Lieber DeCarli ethanol liquid diet and received IS (100 mg/kg) via oral gavage. On the eleventh day of modeling, IS (50 and 100 mg/kg b.w.) was administered to the EISL and EISH groups. In the morning of that day, all animals were gavaged with 31.6% ethanol or isocaloric maltose. Nine hours later, the mice were anesthetized using ether and subsequently euthanized to collect specimens. Liquid feed was obtained from Trophic Animal Feed High-tech Co., Ltd. (Nantong, China). The composition of the Lieber DeCarli control liquid diet was 35% fat, 18% protein, and 47% carbohydrate, whereas the Lieber DeCarli ethanol liquid diet comprised 35% fat, 18% protein, 19% carbohydrate, and 28% ethanol.

### 2.3. Histological and Immunohistochemical Analysis

In this research, animal-derived liver and ileal tissues were fixed, paraffin-embedded, and sectioned to 3 μm thickness. These sections were stained with H&E to assess liver injury and intestinal barrier disruption. Concurrently, frozen hepatic tissues were sectioned into 5 μm slices and subjected to Oil Red O staining for the analysis of lipid accumulation.

The paraffin-embedded ileal samples underwent dewaxing, rehydration, blocking, and overnight incubation at 4 °C with primary antibodies specific to ZO-1 and claudin-1. Following this, secondary antibody incubation was carried out at room temperature for 1 h. The specimens were then reacted with DAB, counterstained with hematoxylin, and dehydrated. For immunohistochemical examination of intestinal barrier proteins, a panoramic scanner (3DHISTECH Ltd., Budapest) was employed, with observations facilitated by CaseViewer software 2.4 (3DHISTECH Ltd., Budapest, Hungary). All histological (H&E, Oil Red O) and immunohistochemical evaluations were performed independently by two investigators who were blinded to the group allocation. Sample slides were labeled with coded identifiers, and quantitative scoring was conducted before the group codes were disclosed so as to minimize potential observer bias.

### 2.4. Serum Biochemical Parameters and LPS Level

AST (aspartate aminotransferase) and ALT (alanine aminotransferase) concentrations in serum, as well as TG (triacylglycerol) and TC (cholesterol) levels in serum and liver tissues, were measured using commercial kits from Nanjing Jiancheng Bioengineering Institute (Nanjing, China) following the manufacturer’s standard protocols. Furthermore, the levels of lipopolysaccharide (LPS) were quantified using enzyme-linked immunosorbent assay (ELISA) kits supplied by Nanjing KeyGen Biotech Co., Ltd., Nanjing, Jiangsu, China.

### 2.5. Tissue Biochemical Analysis

The liver tissue sample (0.1 g) was homogenized in a ratio of 1:9 with phosphate-buffered saline (PBS). Following this, the homogenate underwent centrifugation at 3500 revolutions per minute (rpm) for 10 min at a temperature of 4 degrees Celsius (°C), and the resultant supernatant was collected. Subsequently, the activities of MDA (malondialdehyde), GSH (glutathione), SOD (superoxide dismutase), and CAT (catalase) within hepatic tissues were determined with assay kits obtained from Nanjing Jiancheng Bioengineering Institute, China, strictly following the manufacturer’s protocols.

### 2.6. Gene Expression Analysis (qPCR)

Total RNA was isolated from the tissues using Trizol reagent (Sangon Biotech Co., Ltd., Shanghai, China). Subsequently, cDNA was synthesized by following the protocol outlined in the cDNA Synthesis Mix kit (TaKaRa Bio Inc., Shiga, Japan). For quantitative PCR analysis, the SYBR Premix Ex Taq™ (Tli RNaseH Plus) (TaKaRa Bio Inc., Shiga, Japan) fluorescent dye method was employed. The primer sequences utilized in this study are presented in Table 1. The PCR reaction conditions were as follows: an initial denaturation step at 95 °C for 30 s, followed by 35 cycles of denaturation at 95 °C for 10 s, annealing at 55 °C for 30 s, and extension at 72 °C for 30 s. A melting curve analysis was performed automatically according to the instrument’s settings. As a housekeeping gene, β-Actin served as the reference gene for normalization purposes, and the data were analyzed by means of the 2^−ΔΔCt^ method.

### 2.7. Gut Microbiota Sequencing Analysis

Fecal specimens (50 mg per murine subject) were promptly delivered to Majorbio Bio-pharm Technology Co., Ltd. (Shanghai, China) for genomic analysis. Total microbial DNA was isolated from homogenized samples employing the E.Z.N.A.^®^ soil DNA Kit (Omega Bio-tek, Inc., Norcross, GA, USA) following the standard protocols provided by the manufacturer. Nucleic acid integrity was verified through 1.0% agarose gel electrophoresis (Bio-Rad Laboratories, Hercules, CA, USA), with quantification performed on a NanoDrop^®^ ND-2000 spectrophotometric system (Thermo Fisher Scientific, Waltham, MA, USA). Qualified DNA aliquots were cryopreserved at −80 °C in ultra-low temperature freezers (Thermo Forma, Marietta, OH, USA) for downstream applications.

The hypervariable V3–V4 domains of bacterial 16S rRNA genes were PCR-amplified with degenerate primers 338F (5′-ACTCCTACGGGAGGCAGCAG-3′) and 806R (5′-GGACTACHVGGGTWTCTAAT-3′) using a GeneAmp^®^ 9700 thermal cycler (Applied Biosystems, Foster City, CA, USA). Three independent PCR amplifications were performed for each specimen to ensure reaction reproducibility. Amplicons were size-selected on 2% agarose gels, subsequently purified with the AxyPrep DNA Gel Extraction System (Axygen Biosciences, Union City, CA USA), and quantified via Quantus™ fluorometric analysis (Promega Corporation, Madison, WI, USA).

### 2.8. Western Blot Analysis

After adding lysis buffer containing protease inhibitor and phosphatase inhibitor cocktails to hepatic tissue samples, homogenization and centrifugation were performed to harvest supernatants containing total proteins. Protein quantification was carried out using a commercial BCA kit. Identical quantities of protein were fractionated via SDS-PAGE and blotted onto Polyvinylidene Fluoride Membranes. After protein transfer, the PVDF membrane was placed in a plastic box containing 5% skimmed milk and blocked for 1 h at room temperature, followed by incubation with the corresponding primary antibodies overnight at 4 °C. On the following day, the PVDF membrane was washed five times with TBST buffer solution and incubated with appropriately diluted secondary antibodies for 2 h. Protein bands were visualized using ECL reagents and finally analyzed using ImageJ 1.53t (National Institutes of Health, Bethesda, MD, USA).

### 2.9. Statistical Analysis

All experiments were repeated three times independently, with the results expressed as mean ± standard deviation. SPSS 20.0 software (Inc., Chicago, IL, USA) was used for variance analysis, and Duncan’s multiple range test was adopted to analyze the differences between each experimental group. Statistical significance was defined as *p* < 0.05.

## 3. Results

### 3.1. Dietary IS Ameliorates Alcohol-Induced Hepatic Function Abnormalities

**Figure 1:**
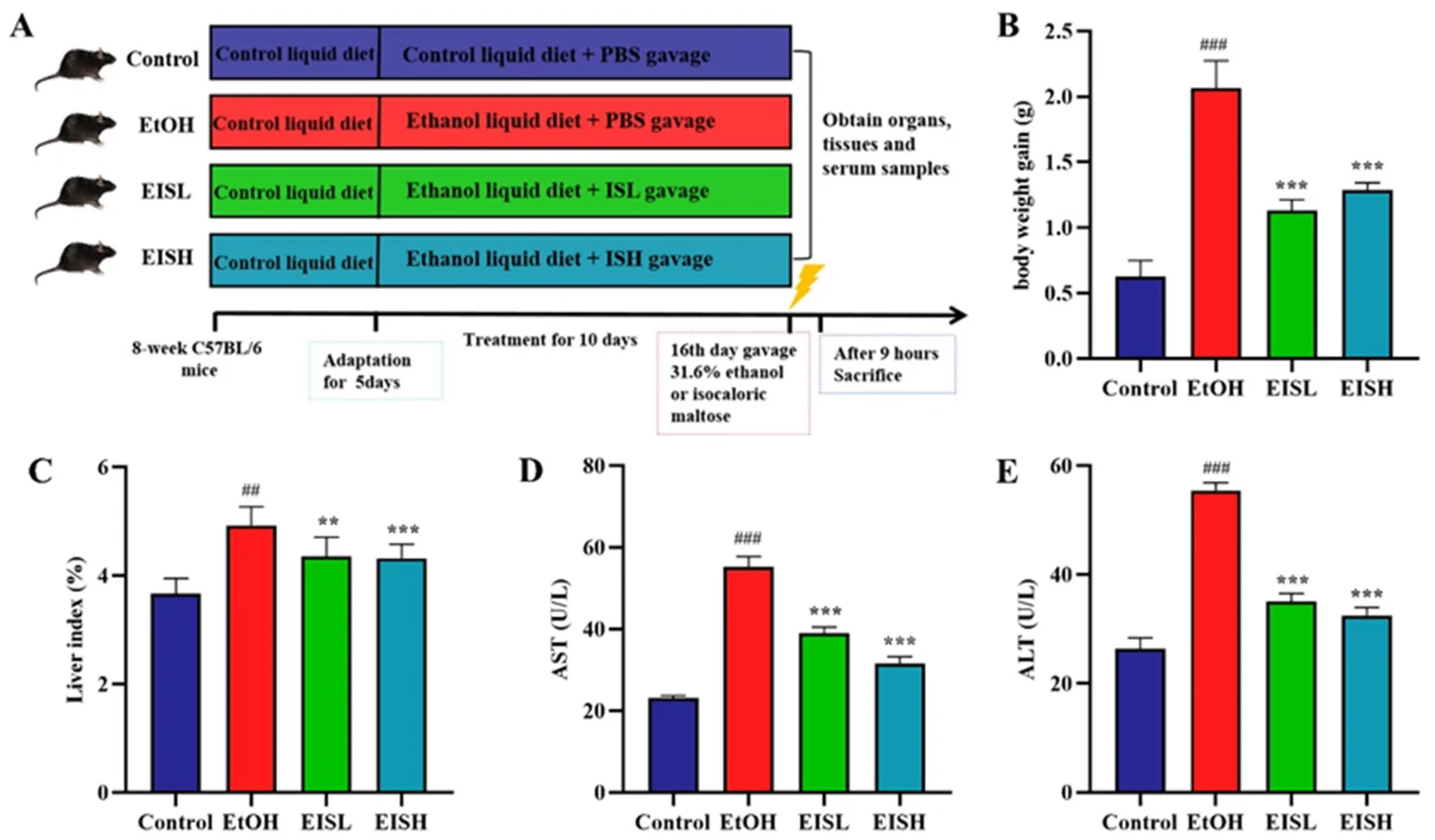
Effect of IS supplementation on body weight gain, liver index, and ALT and AST levels in mice. (**A**) Schematic diagram of animal experiment design. (**B**) Body weight gain. (**C**) Ratio of liver to body weight. (**D**,**E**) Enzymatic activities of serum ALT (alanine aminotransferase) and AST (aspartate aminotransferase). (*n* = 8); Data are presented as the mean ± SD. ## *p* < 0.01, ### *p* < 0.001 versus Control group; ** *p* < 0.01, *** *p* < 0.001 versus EtOH group.

To investigate the ameliorative effects of IS (Insert Specific Substance/Supplement Here) on ALD, an alcoholic liver injury model was first established (Figure 1A), and changes in body weight and liver index were statistically analyzed for each group of mice. The results presented in Figure 1B indicated that there was no significant variation in body weight changes among the groups. Compared to the Control group, the liver index in the EtOH (Ethanol) group was significantly elevated (Figure 1C). This condition was reversed upon supplementation with IS, resulting in a notable decrease in the liver index of the mice. Additionally, serum AST (Aspartate Aminotransferase) and ALT (Alanine Aminotransferase) activities were assayed to explore their impact on liver function. Ethanol consumption severely affected the liver function of the mice, as evidenced by the significant increase in AST and ALT levels (Figure 1D,E). Following the supplementation of the dietary supplement IS, both markers decreased significantly, with the EISH (Ethanol + IS) group approaching normal levels. This also suggests that the addition of dietary supplements can ameliorate liver dysfunction induced by alcohol consumption.

### 3.2. Dietary IS Improves Alcohol-Induced Liver Lipid Accumulation

**Figure 2:**
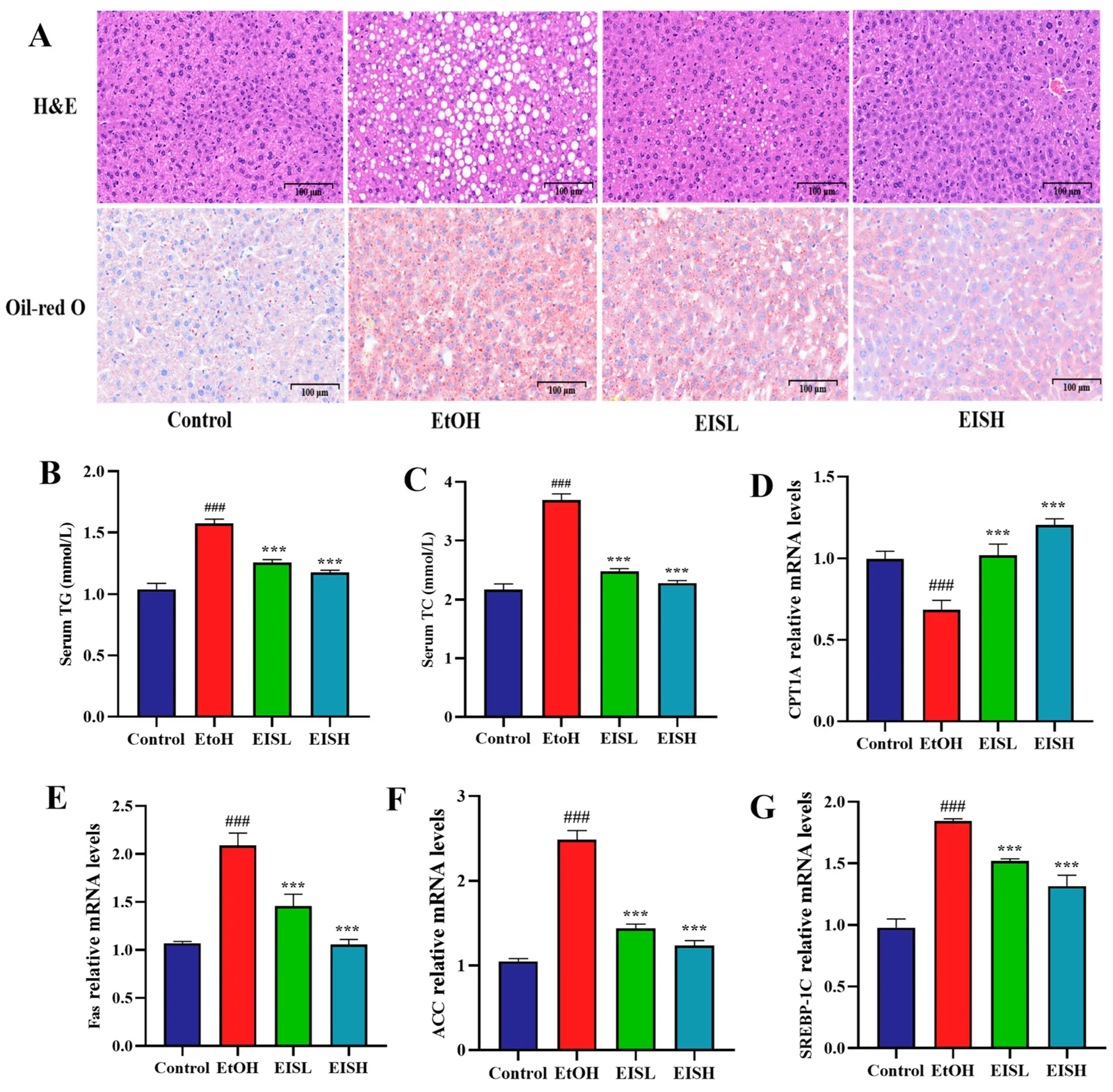
Effect of IS supplementation on hepatic steatosis. Representative images of (**A**) H&E-stained and oil red-O-stained liver tissue sections (scale bar, 100 μm). Serum concentrations of TC (total cholesterol) (**B**) and TG (triglyceride) (**C**). The fold change of mRNA expression in liver tissue (**D**) CPT1A, (**E**) Fas, (**F**) ACC (acetyl-CoA carboxylase), and (**G**) SREBP-1C (sterol regulatory element-binding protein 1C) was determined by qPCR. (*n* = 8); Data are presented as the mean ± SD. ### *p* < 0.001 versus Control group; *** *p* < 0.001 versus EtOH group.

To explore the ameliorative effects of the dietary supplement IS on alcohol-induced dyslipidemia and hepatic steatosis, we conducted oil red O staining and hematoxylin and eosin (HE) staining on mouse liver sections. Based on the staining results, we observed substantial fat accumulation and lipid droplet vacuoles in the livers of the EtOH group, suggesting severe hepatic steatosis resulting from alcohol consumption (Figure 2A). This phenomenon was mitigated after supplementing with a low dose of IS, with the improvement in the EISH group approaching that of the normal group. Consistent findings were also obtained from the measurement of TC and serum TG levels (Figure 2B,C), which were significantly elevated in the EtOH group but significantly reduced in the EISH group. Furthermore, we utilized real-time fluorescent quantitative PCR to examine the expression levels of CPT1A, FAS, ACC, and SREBP-1C in liver tissues (Figure 2D–G). The results indicated that IS increased energy expenditure and lipolysis by enhancing the expression of the fatty acid oxidation-related gene CPT1A. Additionally, IS inhibited the synthesis and accumulation of fat by downregulating the expression of FAS, ACC, and SREBP-1c. Ultimately, these effects lead to an improvement in alcohol-induced hepatic lipid accumulation and steatosis.

### 3.3. Dietary IS Reduces Alcohol-Induced Oxidative Stress in Liver

**Figure 3:**
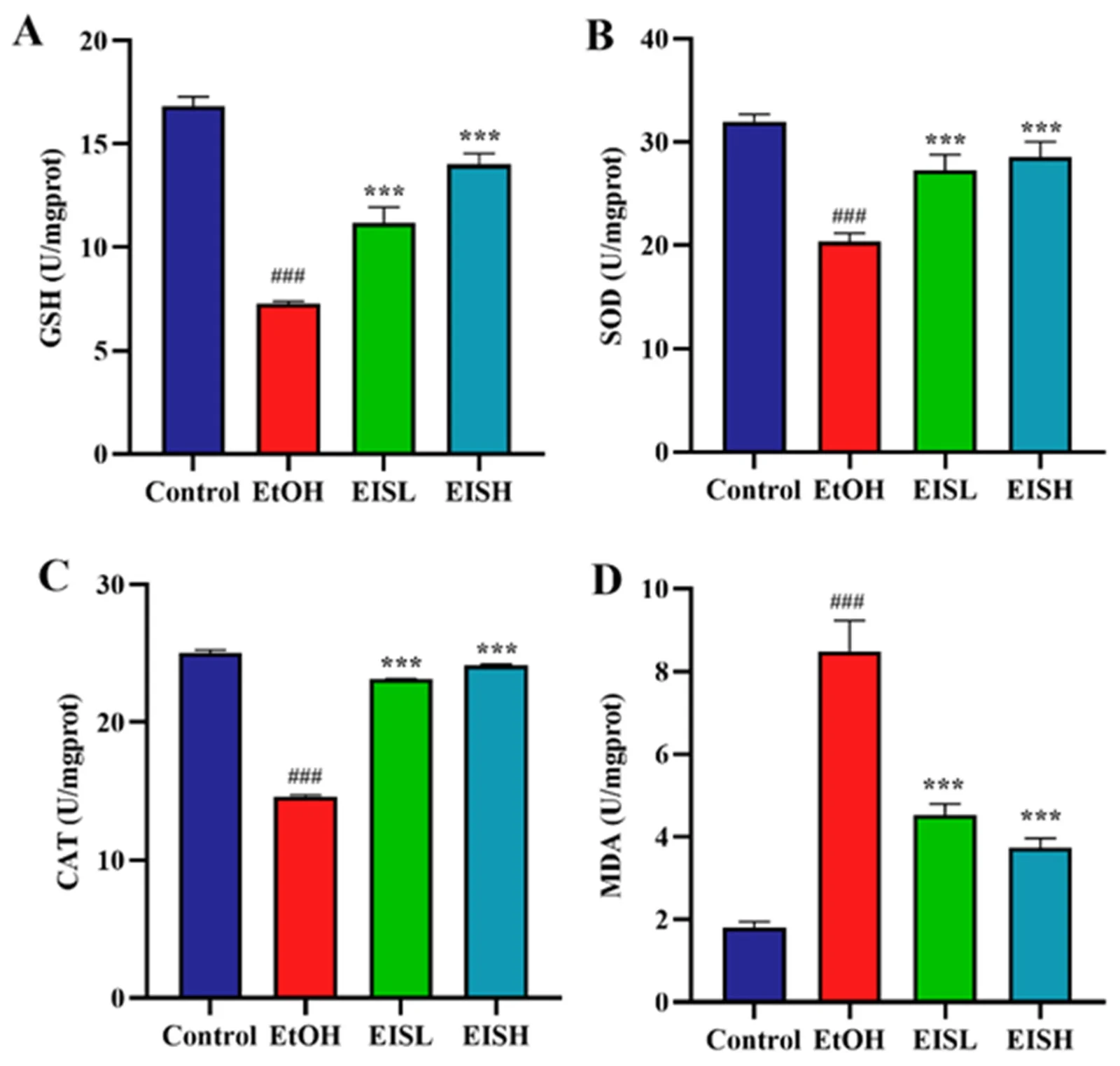
Effect of IS on liver oxidative stress in mice after alcohol consumption. (**A**) Activity of GSH (glutathione) in liver tissue, (**B**) activity of SOD (superoxide dismutase) in liver tissue, (**C**) activity of CAT (catalase) in liver tissue, and (**D**) MDA (malondialdehyde) levels in liver tissue. (*n* = 8); Data are presented as the mean ± SD. ### *p* < 0.001 versus Control group; *** *p* < 0.001 versus EtOH group.

Glutathione peroxidase (GSH), superoxide dismutase (SOD), and catalase (CAT) constitute a vital antioxidant enzyme system within the body, playing indispensable roles in defense against oxidative stress. Malondialdehyde (MDA), as an intermediate product of the deleterious biochemical process of lipid peroxidation, directly reflects the activity level of lipid peroxidation reactions. There exists a close correlation between the health status of the liver and the state of oxidative stress. Consequently, GSH, SOD, CAT, and MDA are frequently employed as key indicators to assess the degree of oxidative stress and cellular damage. In the study, when experimental mice were administered an alcohol-containing diet, their serum levels of SOD, GSH, and CAT enzyme activities exhibited significant declines, while the MDA content surged notably. This result unequivocally demonstrated that an alcoholic diet markedly elevated the level of oxidative stress in the mice. Subsequently, through the introduction of interventions with varying doses of IS, we observed significant increases in the serum levels of SOD, GSH, and CAT enzyme activities in the mice (Figure 3A–C), accompanied by a notable decrease in MDA levels (Figure 3D). Particularly noteworthy is the more pronounced effect observed in the high-dose IS intervention group. In summary, IS has demonstrated significant potential in effectively mitigating oxidative stress induced by alcohol consumption.

### 3.4. Dietary IS Attenuated Alcohol-Induced Intestinal Barrier Damage

**Figure 4:**
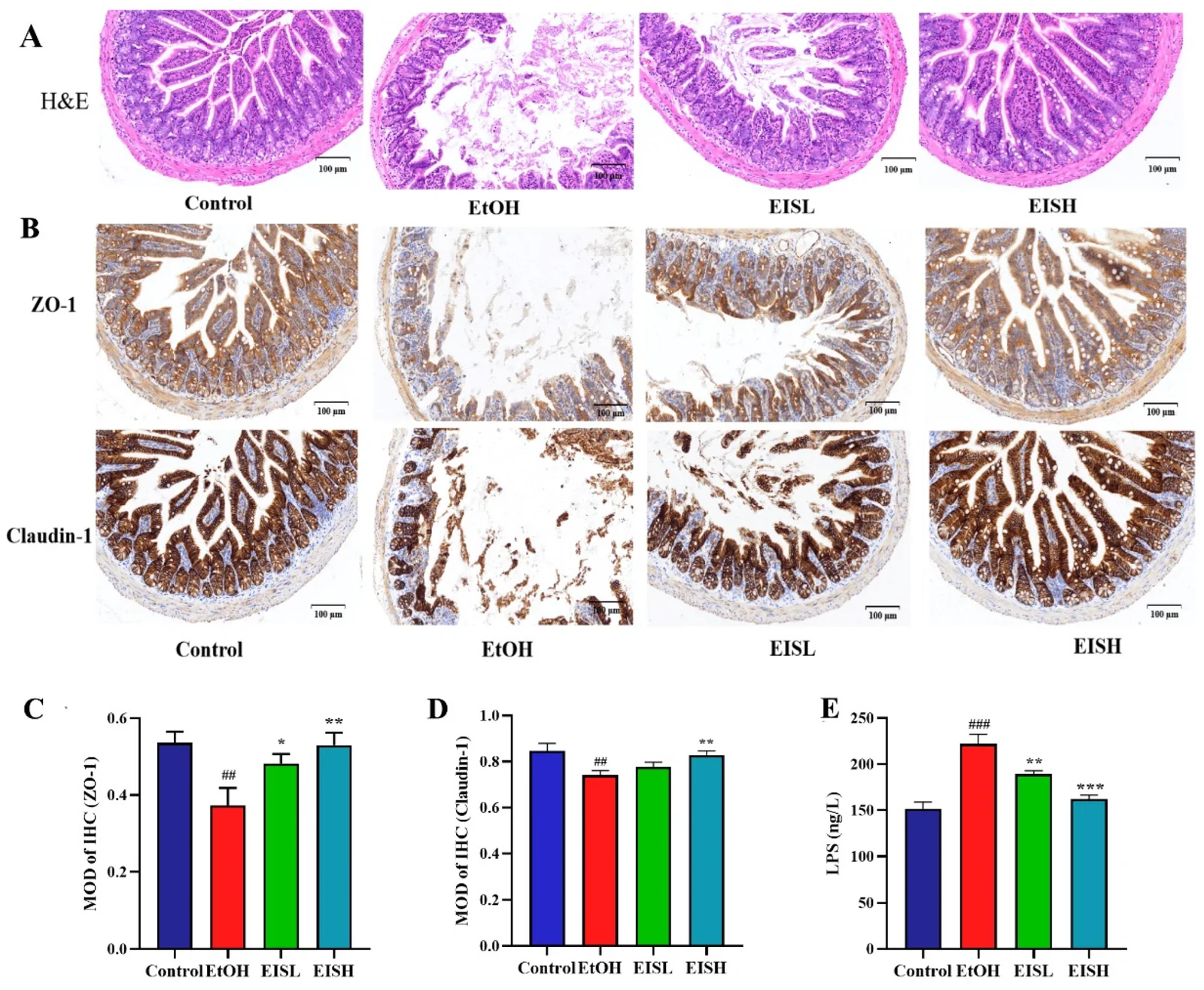
Effect of IS treatment on alcohol-induced endotoxemia and disruption of the intestinal epithelial barrier. concentration. (**A**) H&E staining of ileal tissues (scale bar, 100 μm). (**B**) IHC analysis of ileal TJ protein (ZO-1 and claudin-1) levels (scale bar, 100 μm). (**C**–**E**) Serum LPS (lipopolysaccharide). (*n* = 8); Data are presented as the mean ± SD. ## *p* < 0.01, ### *p* < 0.001 versus Control group; * *p* < 0.05, ** *p* < 0.01, *** *p* < 0.001 versus EtOH group.

Excessive alcohol intake induces severe damage to the intestinal barrier and promotes a significant downregulation of tight junction protein expression. This cascade of reactions leads to increased intestinal permeability, ultimately resulting in a marked elevation of lipopolysaccharide (LPS) levels. Histological analysis of ileal sections reveals clear observations: compared to the Control group unaffected by alcohol, the ileum of mice in the ethanol (EtOH)-treated group exhibits notable atrophy of villi and abnormal changes in intestinal epithelial structure, specifically manifested as sparsity and disarray of peripheral intestinal villi (Figure 4A). In contrast, mice treated with low and high doses of IS exhibit significant improvements in their ileum. Notably, the high-dose IS group demonstrates restored normal morphology of intestinal epithelial cells, alleviating the sparsity and disorganization of peripheral intestinal villi. Following intervention, the ileal mucosa of these mice presents normal histological characteristics, closely resembling those of the Control group. Furthermore, immunohistochemical analysis indicates that the expression levels of ZO-1 and Claudin-1 proteins, key components of intestinal epithelial tight junction (TJ) proteins, are significantly elevated in the high-dose IS group, indicating a restoration to normalcy (Figure 4B). In conclusion, these research data strongly suggest that IS supplementation can effectively mitigate intestinal barrier damage caused by excessive alcohol intake.

### 3.5. Dietary IS Alleviates Endotoxemia and Inflammation Induced by LPS

As a dietary supplement, IS ameliorates intestinal barrier damage induced by excessive alcohol intake and reduces the translocation of gut-derived endotoxin LPS into the bloodstream, thereby decreasing inflammatory responses. Experimental results demonstrate that IS supplementation can significantly lower LPS levels and mitigate the inflammatory responses triggered thereby, including reducing the release of inflammatory mediators and inhibiting excessive activation of immune cells (Figure 4C–E). Consequently, IS exerts a protective effect overall against LPS-induced endotoxemia and its associated intestinal inflammatory responses.

### 3.6. Dietary IS Inhibited Liver LPS/TLR4 Pathway Induction

Elevated intestinal permeability leads to the abnormal translocation of noxious endotoxins from the intestinal cavity into the circulatory system. Subsequently, these endotoxins are delivered to the liver through the gut–liver axis that serves as a connecting bridge. Once in the liver, the endotoxins bind to specific receptors, TLR 4 (Toll-like receptor 4), and this interaction activates a series of downstream signaling pathways, akin to a series of precisely regulated switches, ultimately triggering an inflammatory response within the liver.

**Figure 5:**
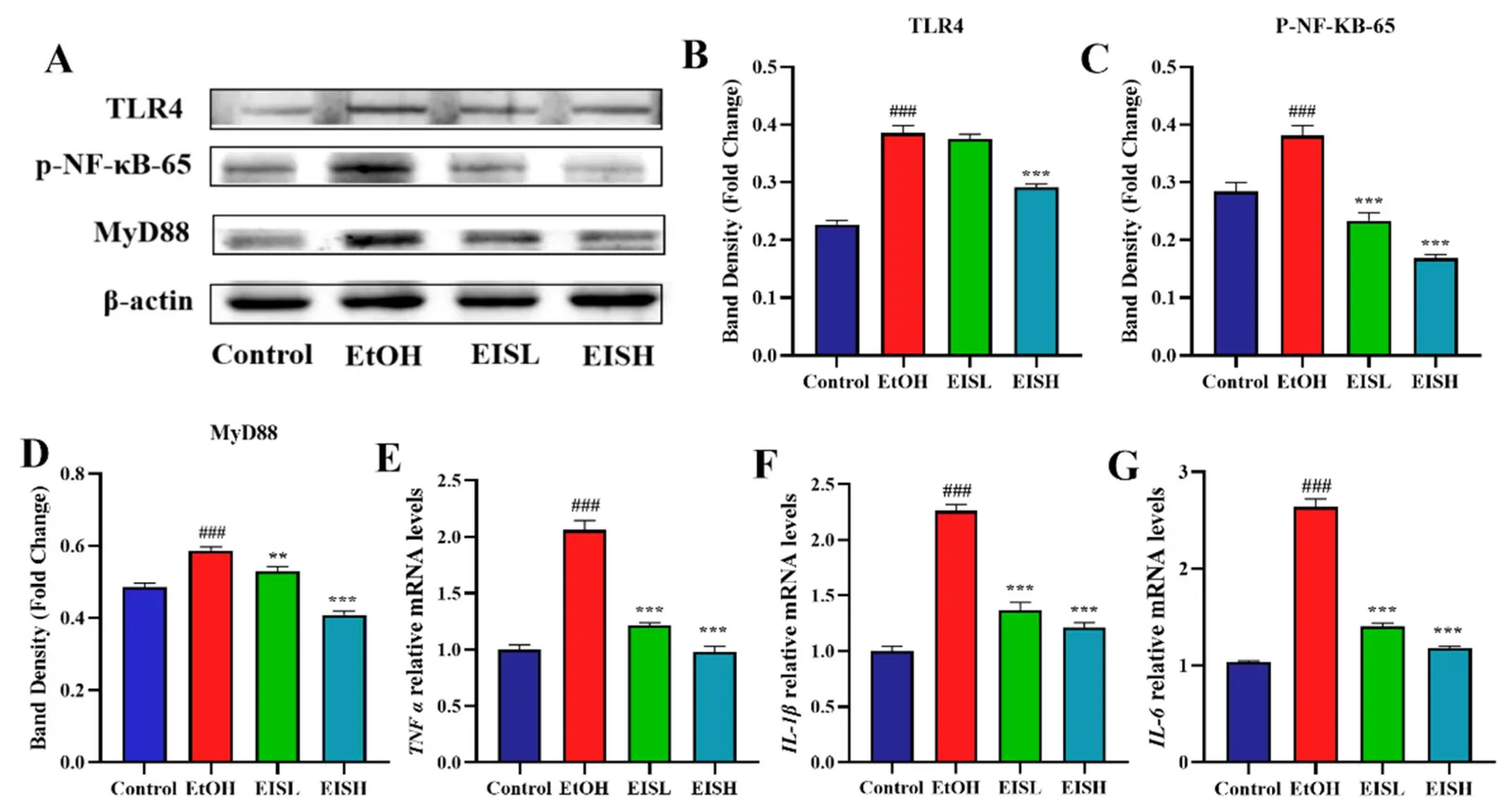
Effect of IS on the TLR4 pathway and liver inflammation in mice after alcohol consumption. (**A**) Western blot analysis of TLR4, MyD88, and p-NF-κBp65 expression levels in liver tissue. Grayscale analysis of (**B**) TLR4, (**C**) p-NF-κBp65, and (**D**) MyD88. The fold change of mRNA expression in liver tissue inflammatory factors (**E**) TNFα, (**F**) IL-1β, and (**G**) IL-6 was determined by qPCR. (*n* = 8); Data are presented as the mean ± SD. ### *p* < 0.001 versus Control group; ** *p* < 0.01, *** *p* < 0.001 versus EtOH group.

In the present study, relative to the Control group that received no special intervention, the hepatic expression levels of TLR4, MyD88, and p-NF-κB p65 in mice treated with alcohol were significantly upregulated, which clearly demonstrated the strong activating effect of alcohol on hepatic inflammatory pathways. (Figure 5A–D). However, when these alcohol-exposed mice were treated with different doses of IS, the expression levels of the aforementioned inflammation-related molecules showed a marked downregulation in a dose-dependent manner. Additionally, in the present study, circulating endotoxin was evaluated by measuring serum LPS levels, whereas the downstream pro-inflammatory mediators (TNF-α and IL-1β) were assessed at the hepatic level (Figure 5E–G), the liver being the principal target organ of the gut–liver axis. The concurrent elevation of serum LPS and hepatic inflammatory cytokines together supports the proposed sequence of intestinal barrier disruption, LPS translocation, and subsequent hepatic inflammatory activation.

In conclusion, IS successfully attenuates the subsequent inflammatory response triggered by endotoxin LPS by effectively blocking the activation of the LPS/TLR 4 pathway in alcohol-induced liver injury. This finding not only reveals the potential mechanism of action of IS in the treatment of alcoholic liver disease but also provides important theoretical and experimental support for the development of novel therapeutic strategies in the future.

### 3.7. Dietary IS Altered the Composition of Microbiota and Reversed Alcohol-Induced Gut Dysbiosis

**Figure 6:**
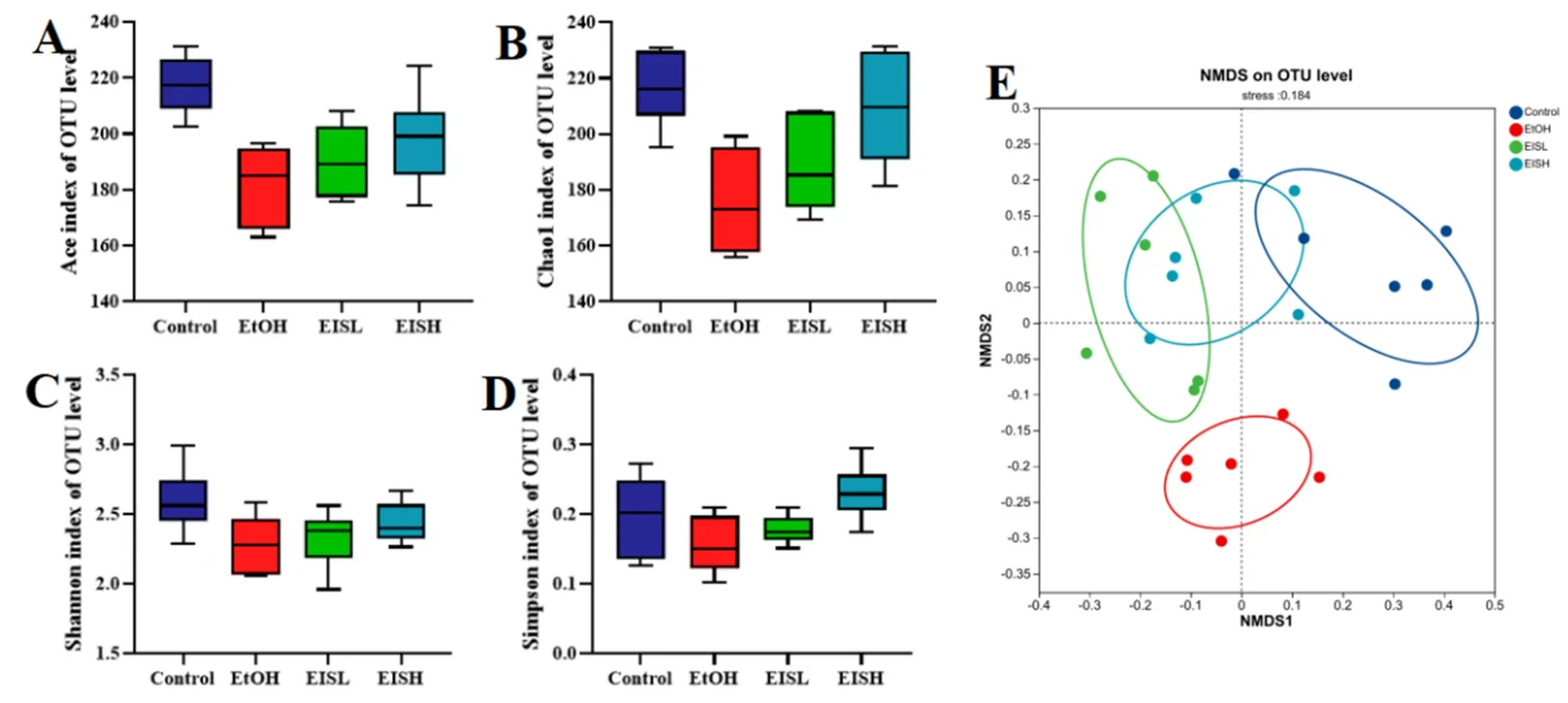
Effect of IS on intestinal microbial diversity. (**A**) ACE, (**B**) Chao1, (**C**) Shannon, and (**D**) Simpson indices of GM. (**E**) NMDS analysis of the *β* diversity. Data represent the mean ± SD of three independent experiments.

In the analysis of α diversity, ethanol consumption significantly decreased the Shannon, Ace, Chao1, and Simpson indices compared to the Control group (Figure 6). Although the Shannon index in the isorhamnetin-supplemented group also decreased, the Simpson index did not differ significantly from that of the Control group. These results imply that ethanol reduces bacterial community richness and diversity, and isorhamnetin supplementation can partially reverse these adverse changes. In contrast, based on β diversity analysis, ethanol intake induced a significant increase in β diversity of the gut microbiota, and isorhamnetin supplementation also restored this change. Furthermore, the NMDS analysis plot showed that the EtOH group and the Control group were clustered separately, whereas the EISH and EISL groups did not exhibit distant clustering from the EtOH group.

**Figure 7:**
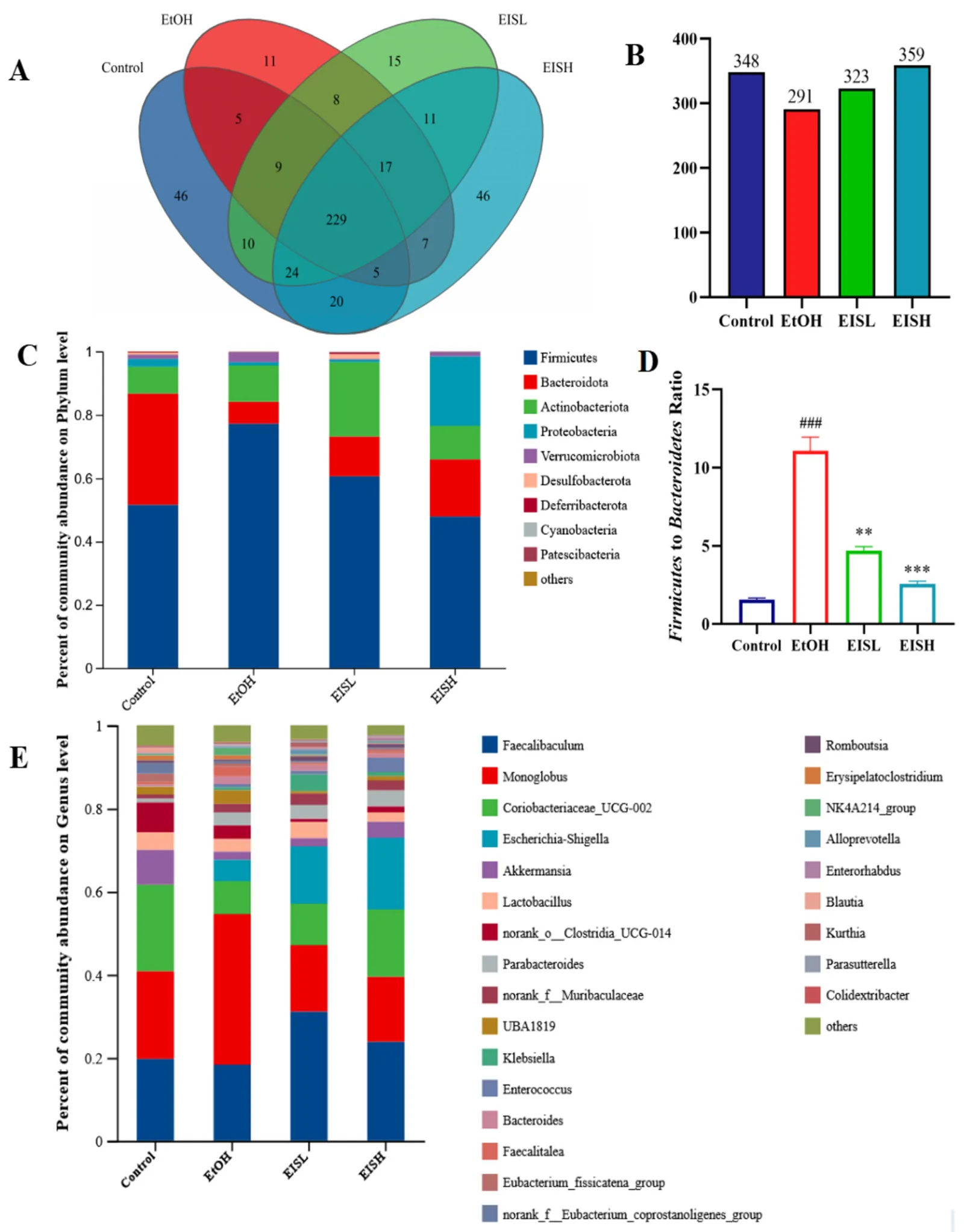
Effects of IS on gut microbiota (*n* = 8). (**A**,**B**) Venn diagram of the OTUs from the gut microbiota. (**C**) Relative abundance of the gut microbiota at the phylum level. (**D**) F/B abundance ratios. (**E**) Relative abundance of the gut microbiota at the genus level. Data are presented as the mean ± SD. ### *p* < 0.001 versus Control group; ** *p* < 0.01, *** *p* < 0.001 versus EtOH group.

The supplement of isorhamnetin effectively alleviated the imbalance of the intestinal microbial community in mice with ALD. To further explore the specific effects of isorhamnetin on the intestinal health status of ALD mice, this study further analyzed the composition of the intestinal microbiota. Through a Venn diagram display, 323 and 359 operational taxonomic units (OTUs) were identified in the low-dose and high-dose isorhamnetin groups, respectively, while the ethanol-fed group EtOH and the control-fed group contained 291 and 348 OTUs, respectively (Figure 7A,B). Analysis based on taxonomy reveals that the gut microbiota of all samples is mainly composed of four phyla: *Firmicutes*, *Bacteroidetes*, *Microsporidia*, and *Proteobacteria* (Figure 7C).

**Figure 8:**
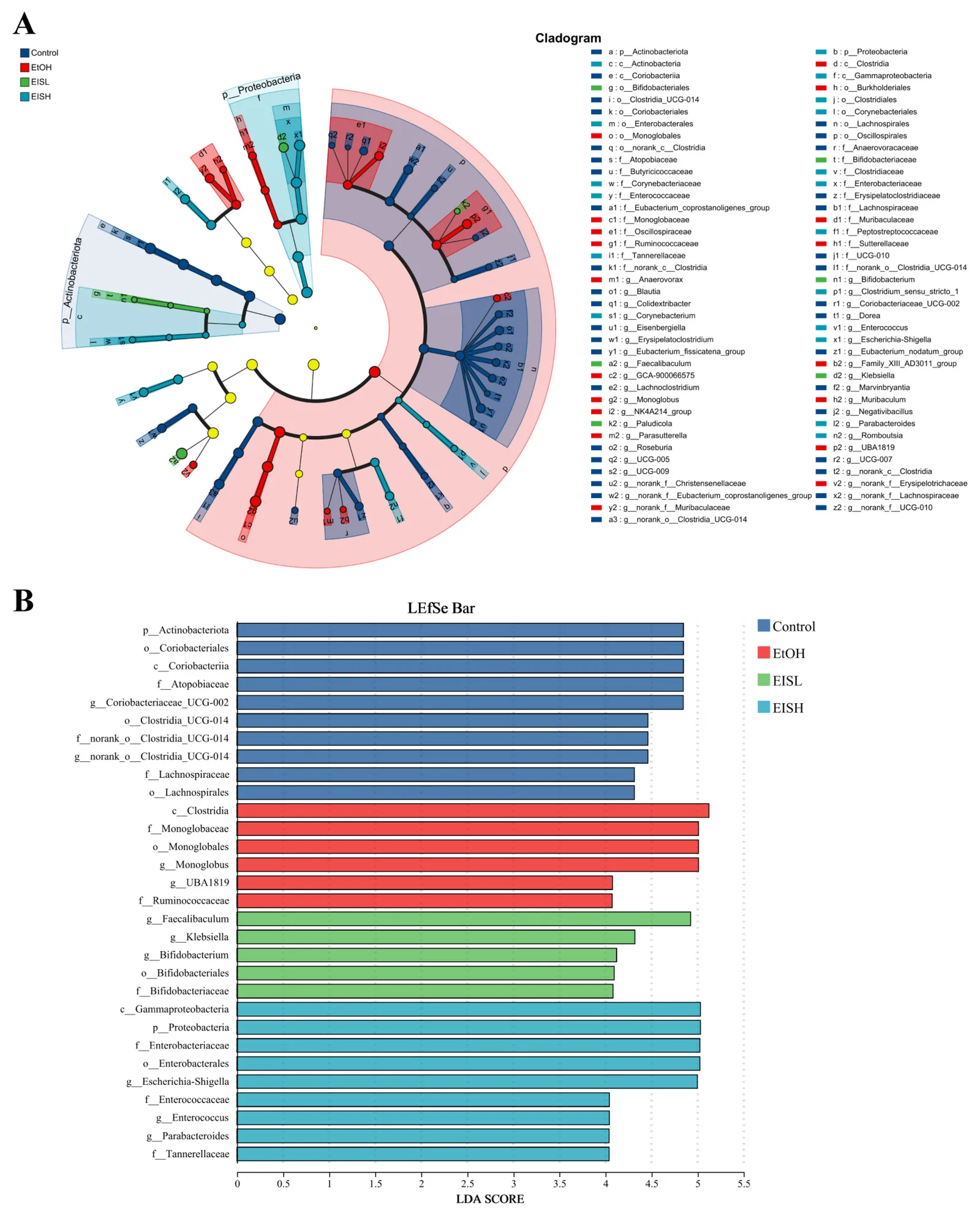
(**A**) LEfSe cladogram indicating that multiple taxa from phylum to genus were differentially enriched in the corresponding groups. The sizes of the dots represent the abundance of microbiota. (**B**) LEfSe bar plot showing the linear discriminant analysis (LDA) scores of the differentially abundant taxa. The length of each bar represents the LDA score, and taxa with higher LDA scores are more significantly enriched in the corresponding group. The Wilcoxon rank-sum test (95% confidence interval) was performed to screen for differential microbiota. LEfSe analysis was performed for differential abundant taxa detected between the EISL + EISH, EtOH, and Control groups (*n* = 8).

In mice receiving ethanol, a reduction in *Bacteroidetes* quantity and an incremental increase in *Firmicutes* quantity were observed, which caused a significant increase in the F/B ratio (*Firmicutes* vs. *Bacteroidetes*) compared to the Control group (Figure 7D). Importantly, the F/B ratio in the isorhamnetin-fed group showed a trend toward the Control group level. Moreover, genus-level analysis results demonstrated that ethanol feeding enhanced the relative abundance of genus M while lowering the relative abundances of genera C and AKK (Figure 7E). However, the treatment with isorhamnetin reversed this trend to some extent. The LEfSe analysis further indicated that Faecalibaculum became the most abundant genus after isorhamnetin treatment. Faecalibaculum has been reported as a short-chain fatty acid (SCFA)-producing genus with potential anti-inflammatory properties and may therefore be associated with beneficial effects on gut and hepatic health (Figure 8). These observations suggest a possible link between the isorhamnetin-induced enrichment of Faecalibaculum and the alleviation of ALD, although the underlying mechanism remains to be further elucidated. In summary, the results of this study indicate that isorhamnetin can effectively improve the ethanol-induced dysbiosis of the gut microbiota.

## 4. Discussion

ALD represents a toxic liver injury induced by chronic and excessive alcohol consumption [17]. The natural course of this disease typically involves fatty liver, alcoholic hepatitis, and alcoholic cirrhosis. During the early clinical phase, it manifests a variety of pathological alterations, including hepatic oxidative stress, inflammatory reactions, lipid metabolism disturbances, and fibrosis [18]. In recent years, the gut–liver axis (GLA) therapeutic strategy for chronic alcohol-induced liver injury has been validated by numerous researchers, aiming to interrupt the vicious cycle of “leaky gut-endotoxin-inflammation” by modulating the interaction between the intestinal microenvironment and the liver [19,20]. Consequently, improving gut microbiota (GM), maintaining intestinal barrier integrity, reducing endotoxemia, and restoring liver metabolic function represent potential novel targets for the treatment of ALD [21].

Due to their antioxidant, anti-inflammatory, and metabolic regulatory properties, flavonoids have been extensively studied and confirmed to exhibit significant hepatoprotective effects [22,23,24]. Numerous studies have demonstrated the liver-protecting capabilities of silymarin, quercetin, apigenin, and other flavonoids [25,26,27]. As a methylated derivative of quercetin, isorhamnetin is more stable and has higher bioavailability than quercetin [28]. Additionally, as a natural dietary component derived from a wide range of plant sources, isorhamnetin holds promise as a dietary supplement for the treatment and prevention of chronic alcoholic liver injury [29]. Existing prior research has confirmed that isorhamnetin possesses the ability to ameliorate steatosis and fibrosis in mouse models of non-alcoholic steatohepatitis [30]. However, the mechanisms and underlying pathways through which isorhamnetin improves chronic alcoholic liver injury via the gut–liver axis remain unclear. Therefore, we investigated the hepatoprotective effects of isorhamnetin at the level of gut microbiota (GM) and its potential mechanisms under conditions of alcohol-induced liver injury.

AST and ALT, as indicators commonly elevated during liver injury, have significant implications for alcoholic liver disease (ALD) [31]. Phenomenologically, supplementary feeding with IS (isorhamnetin) demonstrates a remarkable improvement in alcoholic liver injury. It exhibits favorable effects on liver index as well as liver function indicators AST and ALT. The examination of lipid metabolism indicators, such as serum triglycerides (TG) and total cholesterol (TC), can reflect the disordered state of lipid metabolism in ALD patients [32]. The production of nicotinamide adenine dinucleotide hydrogen (NADH) during ethanol metabolism inhibits the tricarboxylic acid cycle, leading to impaired fat metabolism in the liver [33]. Changes in these indicators are of great value in assessing the degree of fat accumulation in the liver. Meanwhile, liver section analysis can visually demonstrate pathological features such as hepatocyte steatosis, ballooning degeneration, inflammatory infiltration, and fibrosis, serving as an important basis for diagnosing ALD and its staging [34,35]. These indicators provide intuitive insights into the improvement effects of IS supplementation on ALD. Furthermore, IS increases energy expenditure and lipolysis by upregulating the expression of CPT1A, a gene related to fatty acid oxidation. It inhibits fat synthesis and accumulation by downregulating the expression of FAS, ACC, and SREBP-1c. Ultimately, these effects contribute to the improvement of alcohol-induced hepatic lipid accumulation and steatosis. However, to more precisely evaluate the above parameters, further validation at the protein level (e.g., by Western blotting) is warranted to confirm these lipid-metabolic effects of IS.

From a molecular mechanism perspective, the protective effects exhibited by the specific intervention IS (isorhamnetin) against alcoholic liver injury may be associated with the preservation of intestinal barrier integrity. The intestine, as the primary site affected following alcohol exposure, experiences an abnormally increased permeability that has been identified as a core element in the pathogenesis of alcoholic liver disease (ALD). Lipopolysaccharide (LPS), serving as a key biomarker for assessing intestinal barrier integrity, has become an effective means to evaluate the status of intestinal permeability through measurement of its plasma concentration [36]. Existing prior studies have fully confirmed that plasma LPS levels are significantly elevated in patients suffering from ALD and the corresponding animal models [37]. This change not only directly reflects the impaired state of intestinal barrier function but also further exacerbates hepatic steatosis and inflammatory responses, thereby accelerating disease progression. In this study, we conducted an in-depth analysis of mouse models of ALD and found that circulating LPS levels were significantly increased. However, following IS treatment, these mice exhibited a marked decrease in circulating LPS levels. These observations suggest that the hepatoprotective effect of IS may be linked to an improvement in intestinal barrier function, as reflected by reduced circulating LPS. However, as the present study did not directly manipulate gut microbiota or intestinal permeability, this relationship should be interpreted as correlative rather than causal, and further studies employing targeted interventions (e.g., microbiota transplantation or barrier-specific models) are warranted to confirm the underlying causality.

Ethanol, together with its metabolic product acetaldehyde, exerts a disruptive effect on the intestinal epithelial barrier [38]. Both acute and chronic ethanol intoxication can induce histopathological changes like tissue disintegration and separation of intestinal villus epithelial cells [39]. Additionally, ethanol disrupts epithelial tight junctions (TJs), which are major determinants of intestinal barrier integrity. This study, using HE-stained sections and immunohistochemical assays on ileal tissue, demonstrates that IS protects intestinal barrier integrity and significantly elevates the expression levels of ZO-1 and Claudin-1 proteins, key components of TJ proteins in the intestinal epithelium. This further prevents the occurrence of intestinal leakage.

The increased intestinal permeability induces leakage of LPS into the portal vein and subsequently to the liver, where it activates Toll-like receptor 4 (TLR4) on Kupffer cells, ultimately stimulating nuclear factor-κB (NF-κB) [40]. Activation of NF-κB then induces liver inflammation and injury, making the LPS-TLR4-NF-κB pathway critical for the intestine-liver interaction [41]. Our results showed that IS treatment was accompanied by reduced serum LPS together with downregulation of the hepatic LPS/TLR4 signaling components and lower hepatic levels of the pro-inflammatory cytokines TNF-α and IL-1β. It should be noted that, at the circulating level, endotoxemia was assessed specifically by serum LPS, whereas the downstream inflammatory mediators (TNF-α and IL-1β) were measured at the hepatic level, the liver being the principal target organ of the gut–liver axis. The concurrent decrease in serum LPS and hepatic inflammatory cytokines is consistent with, but does not by itself establish, a causal role for the LPS/TLR4/NF-κB axis; these findings therefore support an association between IS treatment and attenuated endotoxin-driven hepatic inflammation rather than a directly demonstrated mechanistic pathway.

The α-diversity indices can quantitatively assess the species richness and evenness of the intestinal microbiota in patients with ALD, and their significant decrease often indicates a simplification of the microbial community structure [42]. In contrast, β-diversity analysis reveals the overall differences in microbiota composition among patients at different disease stages or with different phenotypes, aiding in the identification of characteristic microbiota patterns associated with ALD progression [43]. Supplementation with IS increases the richness and diversity of the intestinal microbiota in mice after alcohol consumption. In α-diversity analysis, the Shannon, Ace, Chao1, and Simpson indices were improved in the IS group. NMDS analysis of β-diversity indicated that IS modified the community characteristics of the intestinal microbiota, forming distinct clusters.

There is a marked correlation between disturbances in the gut microbiome and both the onset and progression of alcoholic liver disease [44]. Metagenomic sequencing analysis has revealed that the gut microbiota of ALD patients exhibits characteristics such as an inverted *Firmicutes*/*Bacteroidetes* ratio, decreased abundance of SCFA-producing bacteria (e.g., *Roseburia* and *Faecalibacterium*), and overproliferation of Gram-negative bacteria. Supplementation with isorhamnetin (IS) can effectively alleviate the imbalance of gut microbiota in ALD mice. Taxonomic analysis reveals that the gut microbiota of all samples is primarily composed of four phyla: *Firmicutes*, *Bacteroidetes*, *Microsporidia*, and *Proteobacteria*. Additionally, compared with the alcohol group, IS supplementation reduces the *Firmicutes*/*Bacteroidetes* (F/B) ratio. In ALD patients, changes in the abundance of Akkermansia (AKK) in the gut microbiota have become a key target in microbiome-gut–liver axis research [45]. Studies have revealed a marked reduction in gut *Akkermansia* abundance in ALD patients, and this decrease may exacerbate intestinal mucosal integrity disruption [46]. Our study found that IS supplementation increased the relative abundance of AKK. As only compositional (relative-abundance) data were obtained in the present study, and neither the functional activity of AKK nor its metabolic output was directly assessed, we refrain from drawing firm conclusions regarding its functional contribution. The observed enrichment of AKK is thus reported as a correlative change, and its potential beneficial role in barrier protection—although supported by previous literature—remains to be functionally validated. Furthermore, Faecalibaculum became the most abundant genus after IS treatment. Faecalibaculum has been reported as a short-chain fatty acid (SCFA)-producing genus with potential anti-inflammatory properties and possible relevance to the management of inflammatory bowel disease. As SCFA concentrations were not measured in the present study, the link between the IS-induced enrichment of Faecalibaculum and the alleviation of ALD is presented as a hypothesis derived from prior literature rather than a directly demonstrated mechanism and warrants further investigation.

To link the microbiota remodeling with the improvement of hepatic injury, the associations between the key differential genera and the inflammatory parameters were further analyzed. IS restored the alcohol-suppressed beneficial genus *Akkermansia*, whose abundance was positively associated with the tight-junction proteins ZO-1 and Claudin-1 and negatively associated with serum LPS, TLR4, and the pro-inflammatory cytokines (TNF-α, IL-1β, IL-6), consistent with its established role in reinforcing the gut barrier and reversing endotoxemia [38,47]. Conversely, the Gram-negative taxa *Escherichia-Shigella* and *Proteobacteria*, as major endogenous LPS sources, showed the opposite trend and were positively correlated with serum LPS and inflammation [48]. These relationships indicate that IS alleviates alcohol-induced hepatic inflammation through the gut–liver axis: by enriching barrier-protective bacteria while suppressing Gram-negative taxa, IS strengthens the intestinal barrier, reduces LPS translocation, and thereby attenuates LPS/TLR4-driven inflammation and liver injury.

## 5. Limitations and Future Perspectives

In this study, several limitations of the present study should be acknowledged. First, isorhamnetin was administered concurrently with ethanol; as flavonoids may interfere with ethanol absorption and modulate its metabolism via CYP2E1, ADH, and ALDH, the current design cannot fully distinguish direct hepatoprotection from a potential modification of ethanol metabolism. A therapeutic design, in which treatment begins only after the liver injury model is established, will be adopted in future studies. Second, the study did not directly manipulate the gut microbiota or intestinal permeability; therefore, the proposed gut–liver axis mechanism should be interpreted as correlative rather than causal, and confirmatory approaches such as fecal microbiota transplantation, antibiotic depletion, or germ-free models are warranted. Third, the microbiota analysis was based on compositional (relative-abundance) data, and functional or metabolic outputs, including short-chain fatty acid concentrations, were not directly measured; hence, the functional consequences attributed to specific taxa (e.g., *Akkermansia* and *Faecalibaculum*) remain to be validated by metabolomic and functional assays. Finally, the findings were obtained in a murine model, and their translational relevance to human ALD requires further clinical investigation. Addressing these aspects in future work will help to more rigorously establish the therapeutic potential and mechanistic basis of isorhamnetin in alcoholic liver disease.

## 6. Conclusions

Collectively, our findings demonstrate that isorhamnetin (IS) ameliorates ethanol-induced hepatointestinal injury through suppression of the LPS/TLR4/NF-κB signaling cascade, resulting in significant attenuation of systemic inflammatory responses. Mechanistically, IS exerts regulatory effects on gut microbiota homeostasis by counteracting alcohol-induced microbial dysbiosis, specifically suppressing pathogenic genera proliferation (*Proteobacteria*, *Enterococcus*, and *Escherichia-Shigella*) while restoring beneficial taxa populations (*Bacteroidetes* and *Parabacteroides*). These therapeutic outcomes are achieved through coordinated mechanisms involving gut barrier reinforcement and hepatic Kupffer cell modulation via the gut–liver axis. The present study substantiates the potential of IS as a novel dietary intervention strategy for alcohol-related liver disease, providing both mechanistic insights and translational potential for functional food development.

## Figures and Tables

**Table 1:** Primer sequences used in qPCR analysis.

Gene	Forward Primer (5′–3′)	Reverse Primer (5′–3′)
*Fas*	TGAATCAGCCCCACGCAGT	CCGAGTCAGTCTTGGAGGACAT
*ACC*	GATGAACCATCTCCGTTGGC	GACCCAATTATGAATCGGGAGTG
*SREBP-1C*	TGACCCGGCTATTCCGTGA	CTGGGCTGAGCAATACAGTTC
*CPT1A*	TGGCATCATCACTGGTGTGTT	GTCTAGGGTCCGATTGATCTTTG
*IL-6*	TAGTCCTTCCTACCCCAATTTCC	TTGGTCCTTAGCCACTCCTTC
*IL-1β*	TTCAGGCAGGCAGTATCACTC	GAAGGTCCACGGGAAAGACAC
*TNF-α*	TCGGGTTCCCATAAGTCAG	CTTGGGATTTGATGCTGGT
*β-actin*	GTGCTATGTTGCTCTAGACTTCG	ATGCCACAGGATTCCATACC

## Data Availability

The data that support the findings of this study are not publicly available due to privacy restrictions but are available from the corresponding author upon reasonable request.

## Supplementary Materials

The following supporting information can be downloaded at: https://www.mdpi.com/article/10.3390/nu18183062/s1, Figure S1: Diagram of IS distribution. Figure S2. Schematic diagram of enzyme-coupled catalysis of rutin to isorhamnetin. Figure S3. LC/MS analysis of isorhamnetin.

## References

[1] Powell E.E., Wong V.W., Rinella M. (2021). Non-alcoholic fatty liver disease. Lancet.

[2] Nontarak J., Rehm J., Rovira P., Assanangkornchai S. (2025). Alcohol-attributable deaths in Thai people from 2015 to 2021 using the comparative risk assessment approach. Alcohol Clin. Exp. Res..

[3] Tsuchida T., Lee Y.A., Fujiwara N., Ybanez M., Allen B., Martins S., Fiel M.I., Goossens N., Chou H.I., Hoshida Y. (2018). A simple diet- and chemical-induced murine NASH model with rapid progression of steatohepatitis, fibrosis and liver cancer. J. Hepatol..

[4] Horn P., Tacke F. (2024). Metabolic reprogramming in liver fibrosis. Cell Metab..

[5] Contreras-Zentella M.L., Hernández-Espinosa L.C., Hernández-Muñoz R. (2025). Oxidative Stress in Liver Metabolic Dysfunction and Diseases, with a Focus on Hepatogenic Diabetes: Effect of Alcohol Consumption. Antioxidants.

[6] Shen H., Zhou L., Zhang H., Yang Y., Jiang L., Wu D., Shu H., Zhang H., Xie L., Zhou K. (2024). Dietary fiber alleviates alcoholic liver injury via Bacteroides acidifaciens and subsequent ammonia detoxification. Cell Host Microbe.

[7] Peng Q., Yang K.M., Hou Q.F., Zheng H.J., Zheng X.M., Xie G.F. (2024). Alleviation of alcoholic liver injury through composite postbiotics regulation of intestinal flora and promotion of bile acid metabolism. Food Biosci..

[8] Nian F., Chen Y., Xia Q., Zhu C., Wu L., Lu X. (2024). Gut microbiota metabolite trimethylamine N-oxide promoted NAFLD progression by exacerbating intestinal barrier disruption and intrahepatic cellular imbalance. Int. Immunopharmacol..

[9] Cui C., Gao S., Shi J., Wang K. (2025). Gut-Liver Axis: The Role of Intestinal Microbiota and Their Metabolites in the Progression of Metabolic Dysfunction-Associated Steatotic Liver Disease. Gut Liver.

[10] Shen N., Wang T., Gan Q., Liu S., Wang L., Jin B. (2022). Plant flavonoids: Classification, distribution, biosynthesis, and antioxidant activity. Food Chem..

[11] Li B.B., Sun S.W., Miao S., Yang L.J., Meng X.Y., Gong K.K., Qi S.Z., Zhang J.J. (2024). Flavonoids from the whole plants of *Limonium sinense* and their anti-inflammatory activity. Nat. Prod. Res..

[12] Zeb M.A., Tu W.C., Li X.L., Xiao W.L. (2024). Phytochemical investigation on *Ginkgo biloba* L. (Ginkgoaceae) and its chemotaxonomic significance. Biochem. Syst. Ecol..

[13] Wei J., Zhao J.M., Su T.T., Li S., Sheng W.J., Feng L.D., Bi Y. (2023). Flavonoid Extract from Seed Residues of *Hippophae rhamnoides* ssp. *sinensis* Protects against Alcohol-Induced Intestinal Barrier Dysfunction by Regulating the Nrf2 Pathway. Antioxidants.

[14] Rana J.N., Gul K., Mumtaz S. (2025). Isorhamnetin: Reviewing Recent Developments in Anticancer Mechanisms and Nanoformulation-Driven Delivery. Int. J. Mol. Sci..

[15] Liu H.-M., Lei S.-N., Tang W., Xun M.-H., Zhao Z.-W., Cheng M.-Y., Zhang X.-D., Wang W. (2022). Optimization of Ultrasound-Assisted Cellulase Extraction from Nymphaea hybrid Flower and Biological Activities: Antioxidant Activity, Protective Effect against ROS Oxidative Damage in HaCaT Cells and Inhibition of Melanin Production in B16 Cells. Molecules.

[16] Zhang H.-Y. (2024). Study on the screening of O-methyltransferase and its biological preparation of tamarixetin and isorhamnetin. Master’s Thesis.

[17] Shi Y.Y., Liu Y., Wang S.J., Huang J.X., Luo Z.Y., Jiang M.S., Lu Y.C., Lin Q., Liu H.H., Cheng N.T. (2022). Endoplasmic reticulum-targeted inhibition of CYP2E1 with vitamin E nanoemulsions alleviates hepatocyte oxidative stress and reverses alcoholic liver disease. Biomaterials.

[18] El-Sehrawy A., Mohammed A.N., Gupta J., Mohammed J.S., Roopashree R., Kashyap A., Janney J.B., Sahoo S., Al-Hasnaawei S., Nasr Y.M. (2025). Combating oxidative stress in non-alcoholic fatty liver disease: From mechanisms to therapeutic strategies. Pathol. Res. Pract..

[19] Xu Q., Zhang R.S., Mu Y., Song Y., Hao N., Wei Y.B., Wang Q.B., Mackay C.R. (2022). Propionate Ameliorates Alcohol-Induced Liver Injury in Mice via the Gut-Liver Axis: Focus on the Improvement of Intestinal Permeability. J. Agric. Food Chem..

[20] Xiao C.Q., Jia R.B., Li X.G., Zhao M.M., Liao W.Z., Zhao S.Q., Xu F.R., Toldrá F. (2024). Musculus senhousei peptides alleviated alcoholic liver injury via the gut-liver axis. Food Funct..

[21] Lok J., Chen C.J., Iannone V., Babu A.F., Lo E.K.K., Vazquez-Uribe R., Vaaben T.H., Kettunen M., Savolainen O., Schwab U. (2025). Advanced Microbiome Therapeutics Accelerate MASLD Recovery by Restoring Intestinal Microbiota Equilibrium and the Gut-Liver Axis in a Mouse Model. J. Agric. Food Chem..

[22] Maleki S.J., Crespo J.F., Cabanillas B. (2019). Anti-inflammatory effects of flavonoids. Food Chem..

[23] Zheng Q.H., Feng K., Zhong W.T., Tan W.J., Rengaowa S., Hu W.Z. (2024). Investigating the Hepatoprotective Properties of Mulberry Leaf Flavonoids against Oxidative Stress in HepG2 Cells. Molecules.

[24] Bayrak S., Gerni S., Ozturk C., Bakan B., Elmas S., Aliyeva A. (2025). In Vitro Evaluation of Flavonoids for Enzyme Inhibition, Antioxidant, Antimicrobial and Anticancer Properties with Molecular Docking Insights. Food Biophys..

[25] Jarosz L.S., Socala K., Michalak K., Bulak K., Ciszewski A., Marek A., Gradzki Z., Wlaz P., Kowalczuk-Vasilev E., Rysiak A. (2025). Subacute exposure to apigenin induces changes in protein synthesis in the liver of Swiss mice. Front. Physiol..

[26] Yin N.N., Pang J., Liu X.Y. (2025). Exploration of the optimal concentration of quercetin liposome nanoparticles for the treatment of liver damage. BMC Pharmacol. Toxicol..

[27] Xiong F., Zhang Y.C., Li T., Tang Y.P., Song S.Y., Zhou Q., Wang Y. (2024). A detailed overview of quercetin: Implications for cell death and liver fibrosis mechanisms. Front. Pharmacol..

[28] Liu L., Barber E., Kellow N.J., Williamson G. (2025). Improving quercetin bioavailability: A systematic review and meta-analysis of human intervention studies. Food Chem..

[29] Zhao S.H., Yang H., Wu H.Z., Li Z.K., Zheng N., Wang J., Zhao H.W., Liu J.F., Sun T.T., Zhang H. (2026). Research progress on the prevention and treatment of alcoholic liver disease with medicinal and edible herbs. J. Ethnopharmacol..

[30] Suttawong N., Witayavanitkul N., Chayanupatkul M., Wanpiyarat N., Werawatganone P., Siriviriyakul P., Werawatganon D. (2025). Gardenia jasminoides fruit extract ameliorates non-alcoholic steatohepatitis with fibrosis by modulating inflammatory and fibrogenic pathways. PLoS ONE.

[31] Lee H.Y., Park Y.M., Shin D.Y., Park K.H., Kim M.J., Yoon S.M., Kim K.N., Yang H.J., Kim M.J., Choi S.C. (2022). Potential Effect of Enzymatic Porcine Placental Hydrolysate (EPPH) to Improve Alcoholic Liver Disease (ALD) by Promoting Lipolysis in the Liver. Biology.

[32] Yuan J., He X.Q., Lu Y., Pu X.H., Liu L.H., Zhang X.J., Liao J.P., Li G.L., Luo Y., Zhang T.W. (2025). Triglycerides/high-density lipoprotein-cholesterol ratio outperforms traditional lipid indicators in predicting metabolic dysfunction-associated steatotic liver disease among US adults. Front. Endocrinol..

[33] Cammisotto V., Valeriani E., Pignatelli P., Violi F. (2025). Nicotinamide Adenine Dinucleotide Phosphate Oxidases and Metabolic Dysfunction-Associated Steatotic Liver Disease. Antioxidants.

[34] Yuri M., Nishimura T., Tada T., Yoshida M., Fujiwara A., Kawata S., Yoshihara K., Yoshioka R., Ota S., Nakano R. (2022). Diagnosis of hepatic steatosis based on ultrasound attenuation imaging is not influenced by liver fibrosis. Hepatol. Res..

[35] Li Y.Y., Zheng T.L., Xiao S.Y., Wang P., Yang W.J., Jiang L.L., Chen L.L., Sha J.C., Jin Y., Chen S.D. (2023). Hepatocytic ballooning in non-alcoholic steatohepatitis: Dilemmas and future directions. Liver Int..

[36] Kong C., Yang M.F., Yue N.N., Zhang Y., Tian C.M., Wei D.R., Shi R.Y., Yao J., Wang L.S., Li D.F. (2024). Restore Intestinal Barrier Integrity: An Approach for Inflammatory Bowel Disease Therapy. J. Inflamm. Res..

[37] Hardesty J.E., Warner J.B., Song Y.L., Rouchka E.C., McClain C.J., Warner D.R., Kirpich I.A. (2023). Resolvin D1 attenuated liver injury caused by chronic ethanol and acute LPS challenge in mice. FASEB J..

[38] Elamin E.E., Masclee A.A., Dekker J., Jonkers D.M. (2013). Ethanol metabolism and its effects on the intestinal epithelial barrier. Nutr. Rev..

[39] Park J.H., Jung I.K., Lee Y., Jin S., Yun H.J., Kim B.W., Kwon H.J. (2021). Alcohol stimulates the proliferation of mouse small intestinal epithelial cells via Wnt signaling. Biochem. Biophys. Res. Commun..

[40] Dumitru A., Tocia C., Badescu A.C., Trandafir A., Alexandrescu L., Popescu R., Dumitru E., Chisoi A., Manea M., Matei E. (2025). Linking gut permeability to liver steatosis: Noninvasive biomarker evaluation in MASLD patients—A prospective cross-sectional study. Medicine.

[41] Meng Z.Q., Zhang Q., Zhang R.R., Liu G.R., Xiang W.L., He Y., Zhang Q., Tang J. (2025). Sinensetin Ameliorates Lipopolysaccharide-Induced Liver Injury by Targeting the TLR4/NF-κB/NLRP3 Pathway and Related Gut-Liver Axis Dysfunction in Mice. J. Agric. Food Chem..

[42] Huang F.R., Lyu B., Xie F.C., Li F., Xing Y.F., Han Z.Y., Lai J.P., Ma J.M., Zou Y.Q., Zeng H. (2024). From gut to liver: Unveiling the differences of intestinal microbiota in NAFL and NASH patients. Front. Microbiol..

[43] Li S.Z., Zhou C., Liu T., Zhang L.H., Liu S.T., Zhao Q., Liu J.K., Zhao W.X. (2024). Causal relationships between the gut microbiota, inflammatory cytokines, and alcoholic liver disease: A Mendelian randomization analysis. Front. Endocrinol..

[44] Mai H.Z., Yang X., Xie Y.L., Zhou J., Wang Q., Wei Y.R., Yang Y.C., Lu D.J., Ye L., Cui P. (2024). The role of gut microbiota in the occurrence and progression of non-alcoholic fatty liver disease. Front. Microbiol..

[45] Wu H.Y., Chen J.J., Guo S.Y., Deng J.H., Zhou Z.M., Zhang X., Qi T.T., Yu F., Yang Q. (2025). Advances in the acting mechanism and treatment of gut microbiota in metabolic dysfunction-associated steatotic liver disease. Gut Microbes.

[46] Shaheen N., Khursheed W., Gurung B., Wang S.H. (2025). Akkermansia muciniphila: A key player in gut microbiota-based disease modulation. Microbiol. Res..

[47] Grander C., Adolph T.E., Wieser V., Lowe P., Wrzosek L., Gyongyosi B., Tilg H. (2018). Recovery of Ethanol-induced *Akkermansia muciniphila* Depletion Ameliorates Alcoholic Liver Disease. Gut.

[48] Rizzatti G., Lopetuso L.R., Gibiino G., Binda C., Gasbarrini A. (2017). Proteobacteria: A Common Factor in Human Disease. BioMed Res. Int..

