# Supplementary material for: Dietary Isorhamnetin Alleviates Alcoholic Liver Injury in Mice by Regulating Gut Microbiota via the Gut–Liver Axis
Source: Nutrients. 2026 Sep 19;18(18):3062. doi: 10.3390/nu18183062 (PMC13609940; doi:10.3390/nu18183062)
Supplement: Supplementary file 1 [file nutrients-18-03062-s001.zip › nutrients-4420831-supplementary.pdf]

Supplementary material

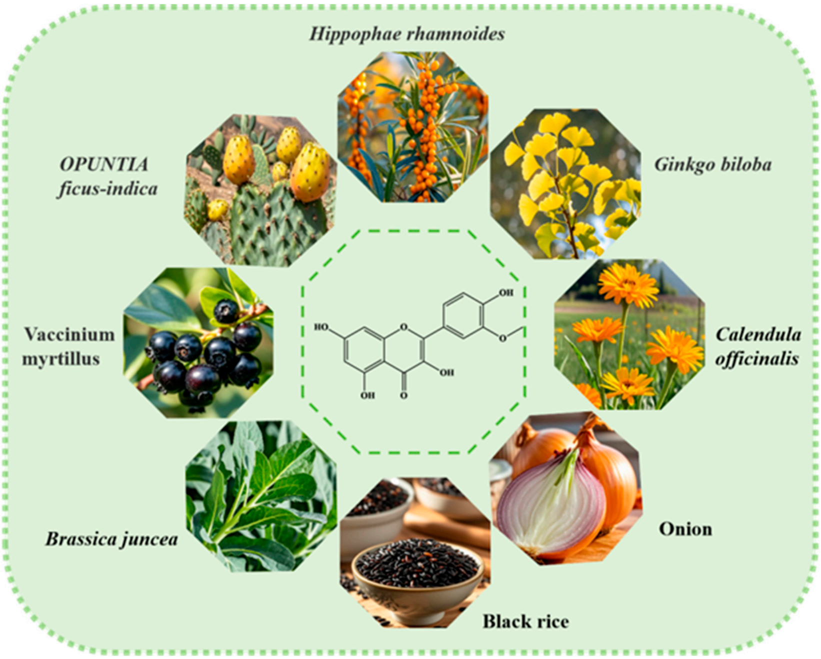

Figure S1. Diagram of IS distribution.

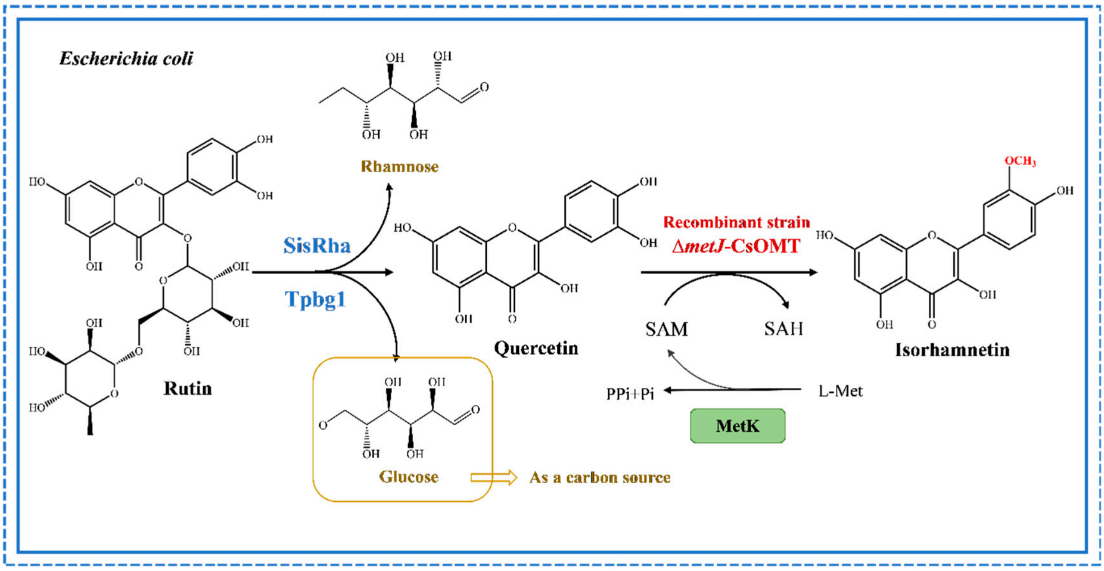

Figure S2. Schematic diagram of enzyme-coupled catalysis of rutin to isorhamnetin

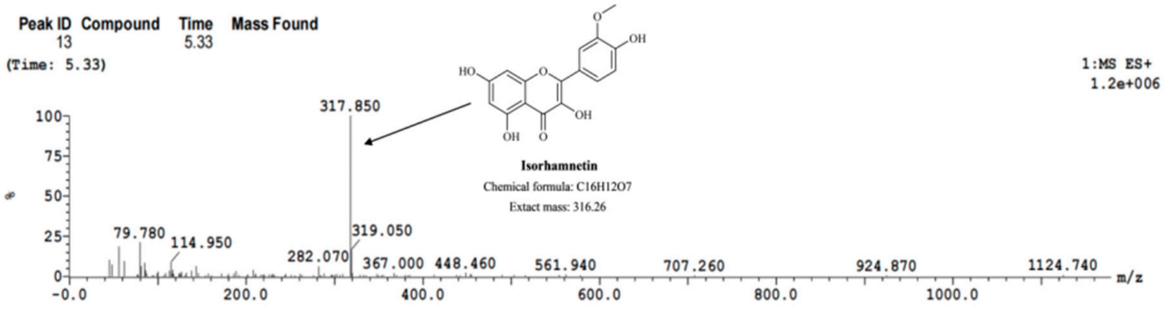

Figure S3. LC/MS analysis of isorhamnetin
